# APOE4 Increases Energy Metabolism in APOE-Isogenic iPSC-Derived Neurons

**DOI:** 10.3390/cells13141207

**Published:** 2024-07-17

**Authors:** Vanessa Budny, Yannic Knöpfli, Debora Meier, Kathrin Zürcher, Chantal Bodenmann, Siri L. Peter, Terry Müller, Marie Tardy, Cedric Cortijo, Christian Tackenberg

**Affiliations:** 1 Institute for Regenerative Medicine, University of Zurich, 8952 Schlieren, Switzerland; 2 Neuroscience Center Zurich, University of Zurich and ETH Zurich, 8057 Zurich, Switzerland; 3 Neurimmune AG, Wagistrasse 18, 8952 Schlieren, Switzerland

**Keywords:** apolipoprotein E (APOE), Alzheimer’s disease, induced pluripotent stem cells (iPSCs), human neurons, energy metabolism, glycolysis, oxidative phosphorylation (OXPHOS), mitochondria

## Abstract

The apolipoprotein E4 (*APOE4*) allele represents the major genetic risk factor for Alzheimer’s disease (AD). In contrast, *APOE2* is known to lower the AD risk, while *APOE3* is defined as risk neutral. APOE plays a prominent role in the bioenergetic homeostasis of the brain, and early-stage metabolic changes have been detected in the brains of AD patients. Although APOE is primarily expressed by astrocytes in the brain, neurons have also been shown as source for APOE. However, the distinct roles of the three APOE isoforms in neuronal energy homeostasis remain poorly understood. In this study, we generated pure human neurons (iN cells) from *APOE*-isogenic induced pluripotent stem cells (iPSCs), expressing either APOE2, APOE3, APOE4, or carrying an *APOE* knockout (KO) to investigate APOE isoform-specific effects on neuronal energy metabolism. We showed that endogenously produced APOE4 enhanced mitochondrial ATP production in *APOE*-isogenic iN cells but not in the corresponding iPS cell line. This effect neither correlated with the expression levels of mitochondrial fission or fusion proteins nor with the intracellular or secreted levels of APOE, which were similar for *APOE2*, *APOE3*, and *APOE4* iN cells. ATP production and basal respiration in *APOE-KO* iN cells strongly differed from *APOE4* and more closely resembled *APOE2* and *APOE3* iN cells, indicating a gain-of-function mechanism of APOE4 rather than a loss-of-function. Taken together, our findings in *APOE* isogenic iN cells reveal an *APOE* genotype-dependent and neuron-specific regulation of oxidative energy metabolism.

## 1. Introduction

Alzheimer’s disease (AD) is the most prevalent age-related neurodegenerative disorder, affecting about 50 million people worldwide [1]. While disease-causing mutations are very rare, genome-wide association studies have identified 90 independent genetic variants associated with AD susceptibility [2]. Among them, the *APOE4* allele represents the strongest risk factor. APOE has three major isoforms, APOE2, APOE3, and APOE4, which differ only in two amino acid residues. While *APOE3/3* is the most common genotype and is defined as risk neutral, the *APOE2* allele is protective but occurs only in 10% of AD patients and 20% of healthy controls. In contrast, the presence of one or two copies of *APOE4* increases the risk by three- or twelve-fold, respectively [3]. In the brain, APOE is mainly expressed by astrocytes, but also microglia and neurons have been shown to secrete APOE. Studies on the selective removal of APOE from the mouse brain indicated that astrocytic APOE makes up 75–80% of total brain APOE while neurons contribute 15–20% [4]. Astrocytes secrete approx. 50–60% of the APOE they produce, while neurons retain most APOE intracellularly and only secrete approx. 10% [5,6] indicating significant differences in APOE biology between neurons and astrocytes. However, if and how neuronal APOE mediated AD pathophysiology remains unclear [7].

APOE has a prominent role in the bioenergetic homeostasis of the brain. The high energy demand of the brain renders it sensitive to changes in energy supply, and alterations in the consumption of glucose as well as deficits in mitochondrial functions are hallmarks of AD [8]. Postmortem studies suggest that reduced brain glucose utilization occurs in patients suffering from AD [9]. Further, FDG-PET analyses in AD patients revealed that cortical glucose hypometabolism is particularly pronounced in *APOE4* carriers [10].

Several studies have been conducted on how APOE4 affects energy metabolism in cell lines or murine primary cultures, with partially contradictory results. However, it is also important to use human cell models to better understand these mechanisms, as cellular functions or expression of several disease-relevant proteins, including APOE, may strongly differ between rodent and human neuronal cells [11]. The use of isogenic lines thereby allows the distinct analysis of APOE effects without bias caused by interpatient genetic variation. Further, we still lack the mechanistic insights into how different APOE isoforms—not only the risk-increasing APOE4 but also the protective APOE2—affect the energy metabolism in human neurons.

In the present study, we show that endogenously produced APOE4 increases mitochondrial energy metabolism in pure *APOE*-isogenic human iPSC-derived neurons (iN cells) but not in the respective iPS cell line. This effect was independent of APOE levels in the different cell lines and did not correlate to levels of mitochondrial fission or fusion proteins. ATP production, as well as basal respiration of *APOE-KO* iN cells were comparable to *APOE2* and strongly differed from *APOE4* iN cells, indicating a gain-of-function mechanism of APOE4 rather than a loss-of-function.

## 2. Materials and Methods

### 2.1. iPS Cell Culture

*APOE*-isogenic iPS cell lines BIONi010-C3 (*APOE-KO*), BIONi010-C6 (*APOE2*), BIONi010-C2 (*APOE3*), and BIONi010-C4 (*APOE4*) [12,13] were purchased from the European Bank of induced pluripotent Stem Cells (EBiSC). iPSCs were cultured on vitronectin (1:25) (100-0763, StemCell Technologies, Vancouver, BC, Canada) coated plates in mTESR+ medium (100-0276, StemCell Technologies), split every 3–4 days, and culture medium was exchanged every other day. For splitting, cells were washed with DPBS, incubated with ReLeaSR (5872, StemCell Technologies) for 4 min at 37 °C with 5% CO_2_, detached in 1 mL mTESR+, and transferred to a new plate.

### 2.2. iN Cell Differentiation

For iPSC differentiation into iN cells, we used the overexpression of Ngn2 as previously described, with slight modifications [14,15]. On day minus one, 500,000 cells per well were seeded as single cells onto new 6-well plates (6 WPs) coated with vitronectin and cultured in mTESR+ medium supplemented with thiazovivin (SML1045, Sigma-Aldrich, St. Louis, MI, USA). Two microliters of each viral construct (TetO-Ngn2-P2A-puromycin, rtTA, and TetO-EGFP) was added to every well. Lentiviral production was performed as previously described [14]. On day zero, the medium was replaced by neural induction medium (N2 1:100, non-essential amino acids (NEAA) 1:100 (11140050, Thermo Fisher, Waltham, MA, USA), doxycycline 2 mg/L (D9891, Sigma, Setagaya, Japan), BDNF 10 ng/mL (450-02, Peprotech, Cranbury, NJ, USA), NT3 10 ng/mL (450-03, Peprotech), and laminin 0.2 µg/mL (L2020, Sigma) in DMEM/F12. Twenty-four hours later, the medium was replaced by fresh medium supplemented with 0.5 mg/L puromycin (P9620, Sigma-Aldrich) to select for transduced cells. On day two, cells were replated to 6 WPs to produce protein or RNA samples, onto 65% nitric acid-pretreated coverslips in a 24 WP for immunocytochemistry (ICC), or to Seahorse XF24 V7 PS plates (100777-004, Agilent, Santa Clara, CA, USA) for seahorse assays. Plates were coated with 100 µg/mL Poly-L-Lysin (P8920, Sigma-Aldrich) and 3.4 µg/mL Laminin (L2020, Sigma-Aldrich). Cells were detached with accutase (A6964, Sigma-Aldrich) and seeded as single cells (750,000 for 6 WP, 100,000 for 24 WP) onto the PLL/Laminin coated plates with neuronal differentiation medium (B27 supplement 1:50 (17504001, Gibco, Billings, MT, USA), Glutamax 1 mM, doxycycline 2 mg/L, BDNF 10 ng/mL, NT3 10 ng/mL, GDNF 10 ng/mL (450-10, Peprotech), CNTF 10 ng/mL (450-13, Peprotech), laminin 0.2 µg/mL, cAMP 0.5 mM (Cay18820-500, Biomol, Hamburg, Germany) in neurobasal medium) and thiazovivin. On day three, the full medium was exchanged to remove thiazovivin. On day five, more medium was gently added (1 mL for 6 WP, 500 µL for 24 WP). Starting from day six, only half of the medium was removed and replaced by fresh medium (1500 µL for 6 WP, 500 µL for 24 WP). Briefly, 2 µM AraC (C1768, Sigma) was added to the medium from day six until the end of the differentiation. Half the medium was exchanged every 3–4 days until day 21. All samples were taken, and experiments were performed on days 21 or 22.

### 2.3. Immunocytochemistry 

iPSCs were fixed one day after replating. iN cells were fixed after differentiation at d21 for 20 min at room temperature (RT) with 4% paraformaldehyde (PFA) (47377.9L, VWR, Radnor, PA, USA) and 4% sucrose (S9378, Merck, Rahway, NJ, USA) in DPBS. Cells were washed three times with PBS for about 5 min at RT and blocked with 10% donkey serum (D9663, Sigma-Aldrich) and 0.1% Triton (X100, Sigma-Aldrich) in DPBS for 1 h at RT (Table 1). This was followed by washing with DPBS, and incubation with primary antibodies diluted in 3% donkey serum and 0.1% Triton in DPBS overnight at 4 °C. After washing the next day, cells were incubated with secondary antibodies diluted in 3% donkey serum and 0.1% Triton in DPBS for 2 h at RT in the dark, washed, and stained with 0.4 ng/µL DAPI (D9542, Sigma-Aldrich) diluted in DPBS for 10 min. Coverslips were mounted with Mowiol (81381, Sigma-Aldrich) on microscope objectives and stored over night at 4 °C protected from light.

### 2.4. RNA Extraction

Cells were harvested with accutase. 1 Mio cells were resuspended in 350 µL RLT buffer and 3.5 µL β-mercaptoethanol (31350-010, Gibco). RNA extraction was performed using the RNeasy Mini Kit (74104, Qiagen, Hilden, Germany) according to the manufacturer’s instructions. The RNA concentrations of the samples were analyzed with the NanoDrop spectrophotometer (Thermo Fisher Scientific) and stored at −80 °C.

### 2.5. Protein Extraction

iPSCs were harvested with accutase, while iN cells were harvested using a cell scraper and resuspended in RIPA buffer supplemented with protease inhibitors (11697498001, Sigma-Aldrich). Due to the high viscosity of iPSC samples, 2 µL of benzonase (E1014, Merck) in a 1 mL sample was added. Four cycles of 30 s of sonication were used to disrupt the cellular membranes. To extract the proteins, samples were centrifuged at 20,000× *g* for 10 min at 4 °C, and the supernatant was finally collected and stored at −20 °C until usage. Protein concentrations (µg/µL) were determined with the Pierce BCA Assay Kit (23252, Thermo Fisher Scientific) according to the manufacturer’s instructions, and the absorption at 562 nm was measured with the Infinite M Nano plate reader (Tecan, Männedorf, Switzerland).

### 2.6. Meso Scale Discovery (MSD) Immunoassay for APOE

The APOE MSD assay (K151AMLR-2, MSD, Rahway, NJ, USA) was performed according to the standard assay protocol 1 from the manufacturer. The plate was coated with 25 µL of biotinylated capture antibody pre-diluted in coating diluent, incubated for 1 h shaking at RT, and washed three times with washing buffer. A serial dilution of the standard was prepared by diluting the 20× stock calibrator in assay diluent, resulting in a standard curve ranging from 750,000 pg/mL to 0 pg/mL. Samples were diluted as follows: iN cell lysates undiluted, iN cell supernatant undiluted, iPSC lysates 1:50, and iPSC supernatant 1:3. Briefly, 1.5 mL of supernatant samples of iN cells was lyophilized before being resuspended in 100 µL RIPA. Thirty microliters of the calibrator standard or sample was added to the coated plate (in duplicate). The plate was incubated at RT for 1 h. After washing 3×, 50 µL of detection antibody solution was added to each well and again incubated shaking for 1 h at RT. After washing 3×, 150 µL of read buffer was added to each well, and the plate was immediately analyzed on an MSD instrument. By using the absorbance of the standard, a standard curve was determined and used to calculate the total APOE levels of the tested samples in relation to the respective dilutions. Measured APOE levels were normalized to total protein concentrations (mg/mL) (see Section 2.5).

### 2.7. Immunoblotting 

Each sample was diluted in RIPA buffer to obtain a concentration of 10 µg and mixed with sample buffer (NP0007, Thermo Fisher Scientific). Samples were denatured for 5 min at 95 °C. Seeblue2 plus protein ladder (LC5925, Thermo Fisher Scientific) and samples were loaded onto 10–20% Tricine SDS-PAGE gels (EC6625BOX, Invitrogen, Waltham, MA, USA) and run at 60 V for 15 min and 100 V for 90 min. Blotting was performed with the Trans-Blot Turbo Mini 0.2 µm nitrocellulose Transfer Pack (1704158, Bio-Rad, Hercules, CA, USA) and the Trans-Blot Turbo Transfer System (1704158, Bio-Rad) at 2.5 A with 25 V for 7 min. Membranes were washed with 0.05% Tween (P1379, Sigma-Aldrich) in PBS and blocked with 5% milk solution (A0830, ITW Reagents, Darmstadt, Germany) in PBS for 1 h at RT shaking. Membranes were then washed three times with PBS-Tween and incubated with primary antibodies diluted in 5% milk solution in PBS-Tween overnight at 4 °C shaking (Table 2). The next day, membranes were washed three times with PBS-Tween and incubated with secondary antibodies diluted in 5% milk solution in PBS-Tween for 2 h at RT in the dark shaking. Membranes were washed three times, developed with one of the ECL selection kits (RPN2232/RPN2235, Cytiva, Marlborough, MA, USA; 32106, Thermo Fisher Scientific), and imaged at the Image Quant 800 (Cytiva). Background subtraction was performed, and protein levels were normalized to the housekeeping proteins GAPDH or β-actin. 

### 2.8. qRT-PCR

Cell-type-specific markers *MAP2*, *TUBB3* and *OCT4* were analyzed. For details on the primers, see Table S1. The data were normalized to the housekeeping marker *GAPDH*. The detection method was SYBR green (1725121, Bio-Rad). For each sample, 5 µL SYBR green, 0.05 µL forward primer, 0.05 µL reverse primer, 2 µL sample, and 2.9 µL nuclease-free water were used. The qRT-PCR protocol was as follows: Separation of the cDNA occurs in an initial hold stage of 10 min at 95 °C, followed by 40 amplification cycles of 15 s at 95 °C and 1 min at 60 °C, and finally one melting curve cycle of 15 s at 95 °C, 1 min at 60 °C, and 15 s at 95 °C.

### 2.9. Seahorse Assay

Using the Seahorse XFe24 (Agilent), the oxygen consumption rate (OCR), the extracellular acidification rate (ECAR), and the proton efflux rate (PER) were measured in living cells without permeabilization. Based on these measurements, all other factors were calculated. Seahorse experiments were performed according to the manufacturer’s instructions. Sensors were preincubated in H_2_O at 37 °C without CO_2_ overnight and changed to calibrant 1 h before the assay. The optimal cell density for each cell type was tested in prior Seahorse experiments. iPSCs were plated at 40,000 cells/well the day before the assay. iN cells were replated at 100,000 cells/well at day 2 of the iN differentiation protocol and analyzed at day 21 or day 22. For each assay, four measurements at baseline and three measurements after drug induction were performed. To avoid detachment of iN cells during the Mito Stress Test, each condition was shorted by one measurement, resulting in three baseline measurements and two measurements after each drug. Each assay type was repeated three times for each cell line and included four background wells for each assay. The Seahorse DMEM medium (103575-100, Agilent) was freshly supplemented with 10 mM glucose, 1 mM pyruvate, and 2 mM L-glutamine. For the ATP Rate Assay, drugs were used at the final concentrations of 1.5 µM oligomycin and 0.5 µM rotenone + antimycin A (ROT/AA). For the Mito Stress Test, drugs were used at the final concentrations of 1.5 µM oligomycin, 1 µM carbonyl cyanide-4 phenylhydrazone (FCCP), and 0.5 µM rotenone + antimycin A (ROT/AA). FCCP titration was performed in earlier Seahorse experiments. After Seahorse assays, cells were immediately fixated with 4% PFA and 4% sucrose in DPBS and stained with 0.4 ng/µL DAPI for 10 min at RT. DAPI was imaged with an inverted fluorescence microscope (Zeiss, Oberkochen, Germany) with 10× magnification. The number of nuclei in each well was automatically analyzed by a macro written in Fiji and used for normalization of the Seahorse data with the Wave Desktop and Controller 2.6 Software (Version 2.6.1).

### 2.10. Data Analysis 

Statistical analysis was performed in Graphpad Prism version 10. The normal distribution was tested using the Shapiro–Wilk normality test and the Kolmogorov–Smirnov test. Outliers were identified with the ROUT test (Q = 1%). Differences between more than two groups were either analyzed by one-way ANOVA for normally distributed data or by the Kruskal–Wallis test for not normally distributed data. One-way ANOVA was followed by a Tukey test for multiple comparisons. A Kruskal–Wallis test was followed by a Dunn’s multiple comparisons test. A *p* value of less than 0.05 was considered significant. Statistical analyses for all graphs are included in the Supplementary Material.

## 3. Results

### 3.1. APOE-Isogenic iPSCs Differentiate into iN Cells and Express Similar Amounts of APOE

**Figure 1:**
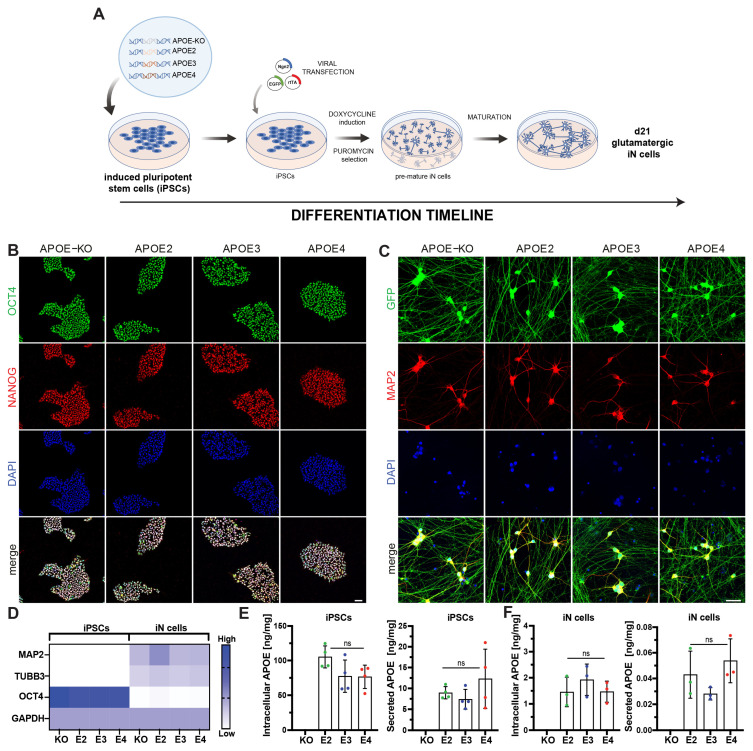
Differentiation of *APOE*-isogenic iPSCs into iN cells. (**A**) Schematic timeline of *APOE*-isogenic iN cell differentiation. (**B**) Representative confocal images of OCT4, NANOG, and DAPI in isogenic iPSCs. (**C**) Representative images of GFP, MAP2 and DAPI in isogenic iN cells. (**D**) Relative gene expression of cell specific iPSC cell (*OCT4*) and neuronal marker (*MAP2* and βIII-tubulin (*TUBB3*) in iPSCs and iN cells, measured by qPCR. (**E**,**F**) Intracellular and secreted APOE protein levels normalized to total protein concentration in iPSCs and in iN cells, measured by MSD. (**E**,**F**): *n* = 3–4 independent samples, one-way ANOVA with Tukey multiple comparison test. ns = not significant; scale bars: 50 µm.

*APOE*-isogenic iPS cell lines BIONi010-C3 (*APOE-KO*), BIONi010-C6 (*APOE2*), BIONi010-C2 (*APOE3*), and BIONi010-C4 (*APOE4*) were used for differentiation into iN cells (Figure 1A). iPSC pluripotency was confirmed by immunostaining for OCT4 and NANOG (Figure 1B). iN cell differentiation was achieved by lentiviral overexpression of neurogenin 2 (Ngn2) [14,15]. iN cells were co-transfected with EGFP to monitor their differentiation status based on cell morphology (Figure S1), showing a network of differentiated neurons at day 21. Mature iN cells were positive for MAP2, demonstrating successful neuronal differentiation (Figure 1C). No differences were observed in marker expression and differentiation potential between the four *APOE*-isogenic lines (Figure 1B,C). qPCR confirmed that iPSCs were positive for OCT4, while iN cells show upregulation of MAP2 and βIII-tubulin (TUBB3) mRNA (Figure 1D). APOE was detected in *APOE2*, -*E3*, and -*E4* iPSCs and iN cells (Figure 1E,F), while no APOE protein was present in *APOE-KO* cells. APOE levels did not significantly differ between the *APOE* lines. In both cell types, iPSCs and iN cells, APOE was mainly localized intracellularly (85–92% intracellular in iPSCs; 96–98% intracellular in iN cells), with overall higher APOE levels per total protein in iPSCs compared to iN cells.

### 3.2. APOE4 iN Cells Show Higher Mito and Glyco ATP Production Than APOE3, -E2 and -KO Cells

**Figure 2:**
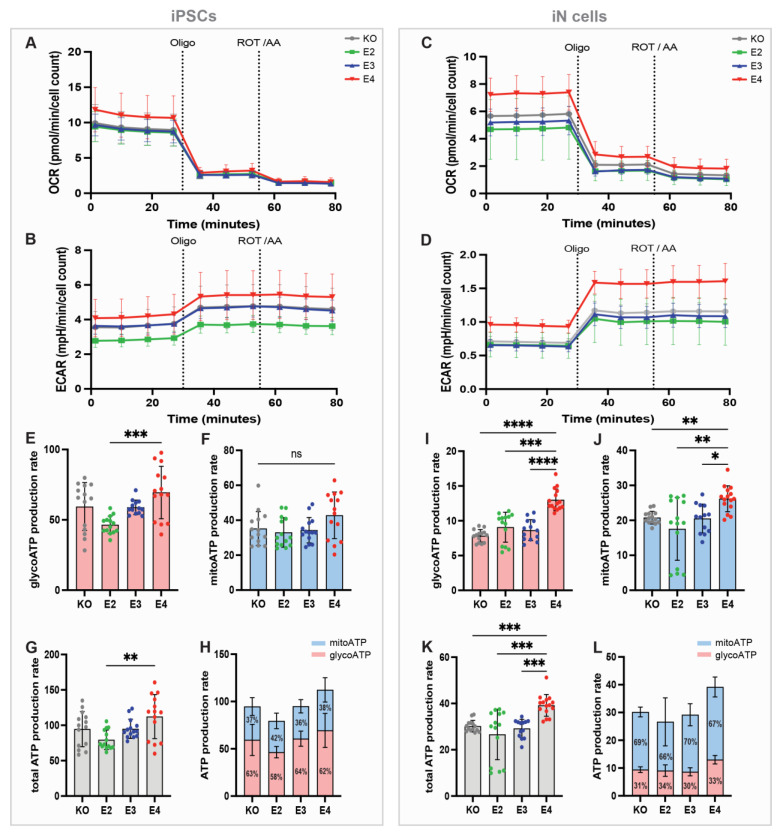
Seahorse ATP rate assay in *APOE*-isogenic iPSCs and iN cells. (**A**) Oxygen consumption rate (OCR) and (**B**) extracellular acidification rate (ECAR) in iPSCs. (**C**) OCR and (**D**) ECAR in iN cells. *APOE-KO* in grey, *APOE2* in green, *APOE3* in blue, and *APOE4* in red. Oligo: oligomycin; ROT/AA: rotenone/antimycin A; (**E**) glycolytic ATP (glycoATP) production rate; (**F**) mitochondrial ATP (mitoATP) production rate; and (**G**,**H**) total ATP production rate in iPSCs. (**I**) GlycoATP production rate; (**J**) mitoATP production rate; (**K**,**L**) total ATP production rate in iN cells. GlycoATP is shown in red, and mitoATP in blue. Kruskal–Wallis test (**F**,**I**–**K**) and one-way ANOVA (**E**,**G**). ns = not significant, * *p* < 0.05, ** *p* < 0.01, *** *p* < 0.001, **** *p* < 0.0001.

*APOE* has been associated with altered brain energy metabolism, while the mechanism and the differential effects of the three major APOE isoforms are still largely unknown. To determine how the *APOE* genotype affects glycolytic and mitochondrial respiration-based ATP production in human cells, a Seahorse ATP rate assay was performed. Oxygen consumption rate (OCR) and extracellular acidification rate (ECAR) were measured four times at baseline and three times after each drug administration in all *APOE* lines in iPSCs (Figure 2A,B) and iN cells (Figure 2C,D). Oligomycin and rotenone/antimycin A were used to inhibit respiratory complex V and I/III, respectively, based on which mitochondrial ATP production could be analyzed. Both *APOE4* iPSCs and iN cells displayed the highest glycoATP production, while no difference was observed between *APOE2*, -*E3*, and -*KO* (Figure 2E,I). No *APOE* genotype effect was found on mitochondrial ATP production in iPSCs (Figure 2F). In contrast, *APOE4* iN cells had significantly higher mitochondrial ATP production compared to iN cells from the other *APOE* lines (Figure 2J), suggesting a cell-type-specific *APOE4* effect. Total ATP production, i.e., the sum of mitoATP and glycolATP, was highest in *APOE4* cells in both cell types, iPSCs and iN cells (Figure 2G,K). However, total ATP production in *APOE4* iPSCs did only significantly differ from *APOE2* iPSCs, while *APOE4* iN cells showed significantly higher total ATP production compared to all other iN cell lines. (Figure 2G,K). As expected, iPSCs gained most of their energy through glycolysis (approx. 60% of total ATP is glycoATP) (Figure 2H), whereas iN cells mainly relied on oxidative phosphorylation (approx. 70% of total ATP is mitoATP) (Figure 2L). 

### 3.3. APOE Genotype Does Not Affect Levels of Mitochondrial Fission and Fusion Proteins in iN Cells

**Figure 3:**
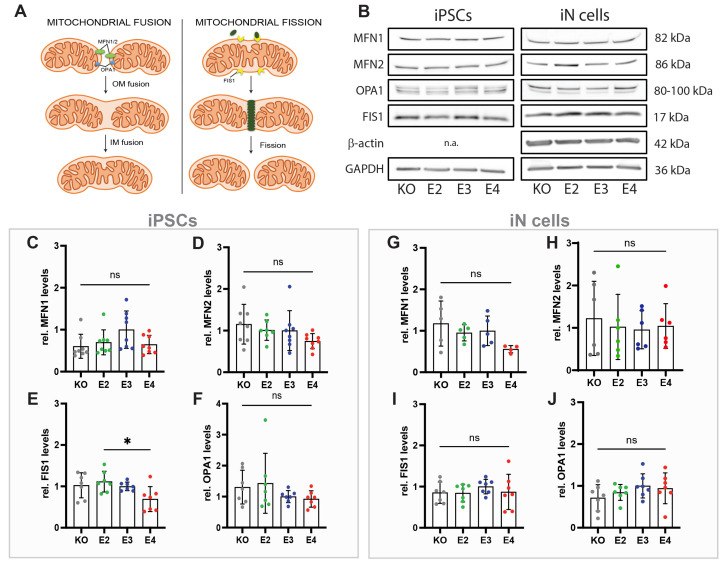
Levels of mitochondrial fusion and fission proteins. (**A**) Schematic overview of mitochondrial fusion and fission markers. (**B**) Representative Western blot images of MFN1, MFN2, FIS1, OPA1, and GAPDH in APOE-KO, -E2, -E3, and -E4 isogenic iPSCs and iN cells. (**C**–**F**) Quantified protein levels of MFN1, MFN2, FIS1, and OPA1 in APOE-KO, -E2, -E3, and-E4 isogenic iPSCs. (**G**–**J**) Quantified protein levels of MFN1, MFN2, FIS1, and OPA1 in *APOE-KO*, -*E2,* -*E3*, and -*E4* isogenic iN cells. All protein levels have been normalized to housekeeping proteins GAPDH or β-actin. Kruskal–Wallis test (**C**,**F**,**H**) and one-way ANOVA (**D**,**E**,**G**,**I**,**J**). ns = not significant, * *p* < 0.05. n.a.: not analyzed; OM: outer mitochondrial membrane; IM: inner mitochondrial membrane.

A key to efficient energy production is maintaining a functional and healthy mitochondrial network. This is ensured by dynamic reshaping events called fusion and fission (Figure 3A). To investigate whether the *APOE* genotype influences these processes in iPSCs and/or iN cells, mitochondrial proteins involved in fusion and fission were analyzed (Figure 3B). No differences between the *APOE* lines were observed for the mitochondrial fusion proteins mitofusin-1/2 (MFN1/2) and OPA1, in iPSCs (Figure 3C,D,F) or iN cells (Figure 3G–J). Mitochondrial fission marker FIS1 was decreased in *APOE4* iPSCs compared to E2 (Figure 3E), but not changed in iN cells (Figure 3I). OPA1 appears as two bands, L-OPA1 and S-OPA1. Both isoforms have been described to cooperate during mitochondrial fusion [16] and were quantified together.

### 3.4. APOE Regulates Mitochondrial Respiration and Respiratory Capacity in a Genotype-Dependent Manner

**Figure 4:**
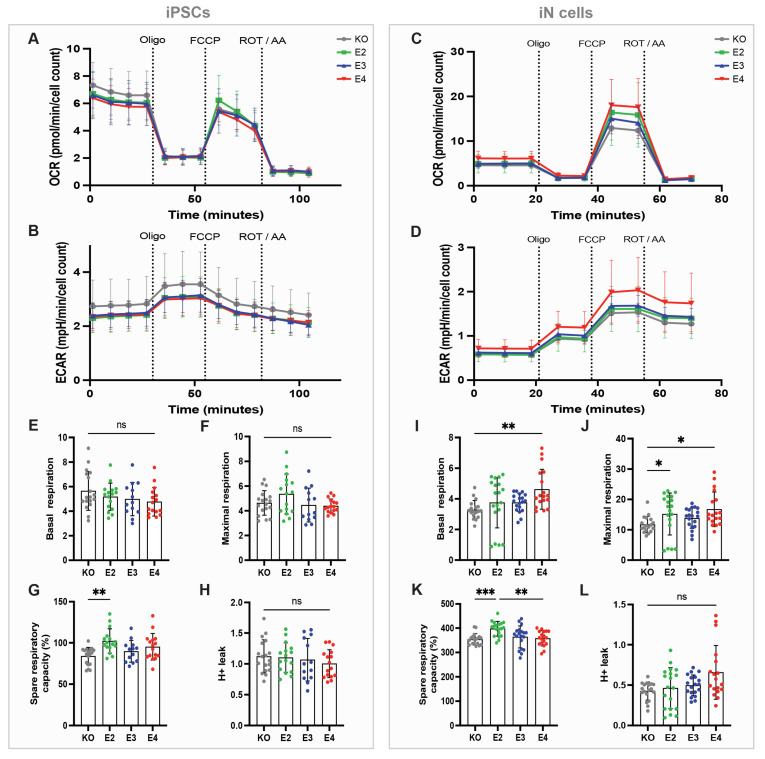
Seahorse Mitochondrial Stress Test in *APOE*-isogenic iPSCs and iN cells. (**A**) Oxygen consumption rate (OCR) and (**B**) extracellular acidification rate (ECAR) in iPSCs. (**C**) OCR and (**D**) ECAR in iN cells. *APOE-KO* in grey, *APOE2* in green, *APOE3* in blue and *APOE4* in red. Oligo: oligomycin; FCCP: carbonyl cyanide-4 (trifluoromethoxy) phenylhydrazone; ROT/AA: rotenone/antimycin A. (**E**) Basal respiration, (**F**) maximal respiration, (**G**) spare respiratory capacity (%) and (**H**) H+ leak in iPSCs. (**I**) Basal respiration, (**J**) maximal respiration, (**K**) spare respiratory capacity (%) and (**L**) H+ leak in iN cells. One-way ANOVA (**E**–**H**) and Kruskal–Wallis test (**I**–**L**). ns = not significant, * *p* < 0.05, ** *p* < 0.01, *** *p* < 0.001.

As we observed an *APOE* genotype effect on mitochondrial ATP production specifically in iN cells but not in iPSCs (Figure 2F,J), we investigated mitochondrial function in more detail. Basal and maximal respiration, as well as mitochondrial capacity and proton leak, were analyzed using the Seahorse Mitochondrial Stress Test. OCR and ECAR were measured at baseline and after the addition of oligomycin, carbonyl cyanide-4 (trifluoromethoxy) phenylhydrazone (FCCP) and ROT/AA in iPSCs (Figure 4A,B) and iN cells (Figure 4C,D). OCR values were used to calculate mitochondrial properties. To induce stress, FCCP was administered, which leads to the collapse of the proton gradient and disruption of the mitochondrial membrane potential, resulting in an uninhibited electron flow through the electron transport chain and maximum oxygen consumption when reaching complex IV. In agreement with our results from the ATP rate assay (Figure 2F), iPSCs lines did not differ in basal and maximal mitochondrial respiration (Figure 4E,F). In contrast, *APOE4* iN cells showed the highest basal as well as maximal respiration (Figure 4I,J), which aligns with the higher mitoATP production shown above (Figure 2J). It should be noted that significant differences were only observed between *APOE4* and *APOE-KO* iN cells. Spare respiratory capacity is an indicator of how well cells can respond to an energetic demand, reflecting cell fitness or flexibility. This was found to be highest in *APOE2* iPSCs and iN cells (Figure 4G,K) compared to the other *APOE* genotypes of the respective cell type. No significant changes in proton leak were observed in any cell line (Figure 4H,L). Taken together, *APOE4* increased mitochondrial respiration in iN cells but not in iPSCs.

## 4. Discussion

In this study, a pure iPSC-derived neuronal culture of *APOE*-isogenic lines was established to study the role of *APOE* in neuronal energy metabolism. We demonstrated that *APOE2*, -*E3*, and -*E4* iN cells express APOE protein, with a small fraction being secreted. This finding is consistent with other studies showing neuronal expression of APOE under physiological conditions [17,18,19,20] with only 10% of neuronally produced APOE being secreted [4,6]. In response to injury, neuronal APOE expression is upregulated as a potentially protective mechanism; however, upregulation of neuronal APOE4 promotes excitotoxic neuronal cell death [17,18,19,20]. *APOE4* neurons produce and secrete less total APOE compared to APOE3, but generate more APOE fragments [21]. Neuronal APOE4 is more neurotoxic in the sense of stimulating neuroinflammation by activating microglia and contributing to signaling pathways of the Aβ and tau pathologies, as well as impairing cholesterol transport by reducing myelin formation [7,22]. However, the exact mechanism by which neuronal APOE4 contributes to AD pathology is still not fully understood. Our study shows that neuronal APOE4 also affects the energy metabolism by increasing mitochondrial and glycolytic ATP production. 

As expected, the primary energy source for iN cells in our study was oxidative phosphorylation (OXPHOS), while iPSCs predominantly rely on glycolysis to meet their energy demands. This aligns with earlier studies showing that the process of reprograming into iPSCs results in a glycolytic signature [23]. Neurons predominantly use OXPHOS for ATP production under basal conditions and just switch to glycolysis when facing increased energy needs [24].

Increased ATP production based on glycolysis was observed in *APOE4* iN cells and iPSCs. These results support the increase in glycolytic enzymes that has been observed in the frontal cortex lysate of AD patients as well as glial cells after treatment with AD plasma [25,26]. Elevated glycolysis has been viewed as a potential compensatory response to mitochondrial dysfunction after exposure to AD plasma, serving as an early indicator of cellular energy deficiency [26,27]. In N2A cells, no glycolytic difference between *APOE2*, -*E3*, and -*E4* cells at an early stage could be observed. However, at higher passages, *APOE4* N2A cells showed impaired glycolysis and glycolytic activity, indicating an age-dependent effect of *APOE4* on their energy metabolism. This effect could also be observed for hexokinase (HK) expression, which is decreased in *APOE4* cells with higher passages, whereas *APOE2* and *APOE3* showed stable expression of HK over time [28]. Qi and colleagues suggested a cell-type-specific APOE effect on glycolysis, showing reduced ATP levels and glycolysis in primary hippocampal neurons and increased glycolytic rates and ATP production in astrocytes [29]. Nonetheless, our study primarily analyzed glycolytic ATP production, underscoring the need for further research to investigate the impact of *APOE4* on neuronal glycolytic metabolism in the future.

We show that APOE regulates mitochondrial respiration and respiratory capacity in a genotype-dependent manner, with the highest basal and maximal respiration in *APOE4* iN cells compared to *APOE-KO* and the highest spare respiratory capacity in *APOE2*. This aligns with earlier studies showing increased oxidative stress mechanisms and ROS production as well as upregulation of mitochondrial respiratory complexes in iPSC-derived neurons from sporadic AD patients, even in the absence of changes in Aβ or tau and p-tau levels [14]. Mitochondrial and glycolytic impairments had already been linked to AD before [30]. *APOE4* carriers show mitochondrial dysfunction in brain areas associated with AD even before the onset of amyloid or tau pathology or cognitive changes, indicating that mitochondrial dysfunction is involved in early AD pathology [31]. However, contrary to our findings of mitochondrial upregulation and enhanced ATP production in *APOE4* cells, some studies indicated mitochondrial downregulation and decreased ATP production. *APOE4* primary hippocampal neurons from mice showed lower maximal respiration and spare respiratory capacity ratios, as well as lower mitochondrial membrane potential, reduced expression of complex subunits, and reduced ATP levels. [29]. Neuronal APOE4 expression reduced the levels of mitochondrial respiratory complex subunits compared to *APOE3* neurons [32,33]. APOE4 expression in N2A cells decreased the enzymatic activity of complex IV [32]. However, these studies have primarily relied on animal models or cell lines, which may have different physiological and metabolic properties compared to human neurons, resulting in different outcomes. Further, Chen and colleagues observed changes in respiratory chain complexes in neurons but not in astrocytes, indicating that mitochondrial dysfunction based on APOE4 expression is neuron-specific [32]. Neuron-specific proteolysis of APOE4, resulting in APOE4 fragments in the cytosol, caused toxic effects such as tau phosphorylation, alterations in cytoskeleton, and mitochondrial dysfunctions [34,35].

Mitochondria are highly dynamic organelles undergoing regular fission and fusion cycles. Mitochondrial fusion is particularly important in respiratory active cells, such as neurons [36]. Therefore, we analyzed proteins involved in fusion and fission, but did not observe any *APOE*-dependent effects in iN cells. This suggests that mitochondrial dynamics are not affected by *APOE*, independent of further mitochondrial functions, including the electron transport chain (ETC). This is in agreement with a previous study showing altered levels of ETC complexes in iN cells from AD patients in the absence of fusion and fission alterations [14], suggesting that human iN cell fusion and fission are stable mechanisms that are not affected by other mitochondrial dysfunctions. In contrast, APOE-dependent alterations in mitochondrial fusion/fission protein levels have been shown in other studies; however, those were contradictory. MFN1, MFN2, OPA1, and FIS1 levels were decreased in the brains and neurons of AD patients and *APOE4* carriers [37,38], whereas *APOE4* N2A cells expressed higher levels of fission and fusion proteins than *APOE3* N2A cells [33]. These studies suggest that additional mechanisms, beyond the *APOE* genotype, may play a role in regulating mitochondrial fusion and fission in AD. 

In general, many contradictory results on the impact of APOE on energy metabolism have been published. Some studies found that APOE4 reduced glycolysis [28,39], whereas others observed a shift from oxidative to glycolytic metabolism in *APOE4* cells with higher glycolytic activity in *APOE4* than *APOE3* [40,41,42]. Our study showed enhancement of both oxidative as well as glycolytic metabolism. The variations may be attributed to discrepancies across studies encompassing differences in cell types, cell species, and the time point of analysis.

In addition to *APOE4* and *APOE3* cells, our study included *APOE2* and *APOE-KO* iN cells. We did not observe differences in intracellular or secreted APOE levels between the isogenic lines, but generally higher APOE levels per total protein level in iPSCs than in iN cells. This indicates that the APOE4-induced increase in neuronal energy metabolism is independent of the levels of APOE4 protein. When comparing *APOE-KO* cells to the other *APOE* cell lines, we observed that *APOE-KO* iN cells have a similar response as *APOE2* and *APOE3* but not as *APOE4*, concluding that *APOE4* rather displays a gain-of-function than a loss-of-function mechanism. This was also observed in astrocytes from the same iPSC lines, where *APOE-KO* astrocytes showed a similar response in glutamate and Aβ uptake, cholesterol, and lipid metabolism, as well as an inflammatory response to *APOE2* but not to *APOE4* astrocytes [13]. This is also consistent with the study of Chemparathy and colleagues showing the protective function of *APOE* loss-of-variants in healthy individuals and AD patients [43].

Especially the time point of analysis seems to be a critical variable between the studies. On the first view, our data of increased glucose metabolism in *APOE4* iN cells contradict the findings of a reduced metabolism (hypometabolism) observed in *APOE4* carrier. However, previous studies have already linked *APOE4* to increased (hyper-)metabolism in young individuals. Studies in young adults, before the onset of a pathology, showed that *APOE4* carriers display hypermetabolism and hyperactivity in distinct brain regions, such as the hippocampus, entorhinal cortex, and cortical regions [44,45,46]. Further, several studies using *APOE4* KI mouse models, that do not yet show features of neurodegeneration, observed a hypermetabolism caused by *APOE4* [47,48]. Therefore, we suggest that hypermetabolism is an early feature that may be triggered by *APOE4*, especially before the onset of AD pathology. However, with age, metabolic activity switches to hypometabolism. The reason may be that an early hypermetabolism drives AD pathology, including amyloid production and aggregation as well as tau phosphorylation and accumulation, which leads to neurodegeneration [48]. Subsequently, this neurodegeneration results in a hypoactive state once the disease process has reached a critical stage. As iPSC models are considered to represent very young cells and are supposed to be models of early disease or even before disease onset, we observed the early hypermetabolism instead of hypometabolism. This is further supported by a recent study showing that APP KI mice display mitochondrial hypermetabolism before the onset of any pathology. Upon increasing pathologies, the brain shifted to a state of hypometabolism [49]. In a recent comment, Sercel and colleagues wrote that it was long thought that mitochondrial diseases are linked to ATP deficiency, but that several studies now rather link mitochondrial and OXPHOS deficiency to hypermetabolism [50]. One potential mechanism could be the upregulation of various biological processes and stress responses to compensate for OXPHOS deficits, ultimately resulting in hypermetabolism.

## 5. Conclusions

It is important to recognize that this study only scratches the surface of understanding human neuronal energy metabolism in the context of *APOE* genotypes. While the observed mitochondrial and glycolytic abnormalities are significant, there is a need for further investigations into the mechanistic basis of the observed *APOE4*-mediated increase in energy production. Especially the comparison between early and late time points of analysis should be the focus of future research. Further, this study was based on glutamatergic neurons. However, it has been shown that inhibitory neurons express higher levels of APOE4, and the proportion of inhibitory interneurons could be correlated with AD disease progression [4,34,48], highlighting the need for including a wider range of neuronal subtypes in future studies. It will also be interesting to investigate the *APOE* effect on iPSC-derived astrocytes from the same isogenic iPSC lines to discriminate between neuronal and astrocytic effects. More studies are necessary to further untangle the molecular pathways and signaling cascades involved in *APOE4*-induced mitochondrial and glycolytic dysfunction to understand the pathophysiology underlying neurodegenerative diseases such as AD.

## Figures and Tables

**Table 1:** List of antibodies used for ICC.

**Primary Antibody**	**Producer**	**Cat. No.**	**Dilution**
Anti-Oct4	Cell Signaling	2890S	1:200
Anti-Nanog	Thermo Fisher	14-5768-82	1:100
Anti-MAP2	Synaptic systems	188011	1:500
**Secondary Antibody**	**Producer**	**Cat. No.**	**Dilution**
Dk-α-ms-cy3	Jackson ImmunoResearch	715-165-151	1:1000
Dk-α-rb-cy5	Jackson ImmunoResearch	715-175-152	1:1000
Dk-α-rb-Alexa647	Jackson ImmunoResearch	715-605-152	1:250

**Table 2:** Antibodies used for Western blot.

**Primary Antibody**	**Producer**	**Cat. No.**	**Dilution**
Anti-MFN1	Cell Signaling	14739S	1:250
Anti-MFN2	Cell Signaling	11925S	1:1000
Anti-FIS1	Abcam	ab15686	1:10,000
Anti-OPA1	Cell Signaling	67589S	1:2000
Anti-GAPDH	Meridian	H86504M	1:5000
Anti-β-actin	Abcam	Ab6276	1:20,000
**Secondary Antibody**	**Producer**	**Cat. No.**	**Dilution**
Dk-α-ms-peroxidase	Jackson ImmunoResearch	715-035-151	1:5000
Dk-α-rb-peroxidase	Jackson ImmunoResearch	111-035-144	1:5000

## Data Availability

The data that support the findings of this study are available from the corresponding author upon request. All statistical analyses are found in the Supplementary Data.

## Supplementary Materials

The following supporting information can be downloaded at: https://www.mdpi.com/article/10.3390/cells13141207/s1; Figure S1: iN cell differentiation over time. Table S1: List of qPCR primer. Excel sheet: Statistical analyses for all bar graphs in the manuscript.
