# Supplementary material for: APOE4 Increases Energy Metabolism in APOE-Isogenic iPSC-Derived Neurons
Source: Cells. 2024 Jul 17;13(14):1207. doi: 10.3390/cells13141207 (PMC11274733; doi:10.3390/cells13141207)
Supplement: Supplementary file 1 [file cells-13-01207-s001.zip › cells-3077110-supplementary.pdf]

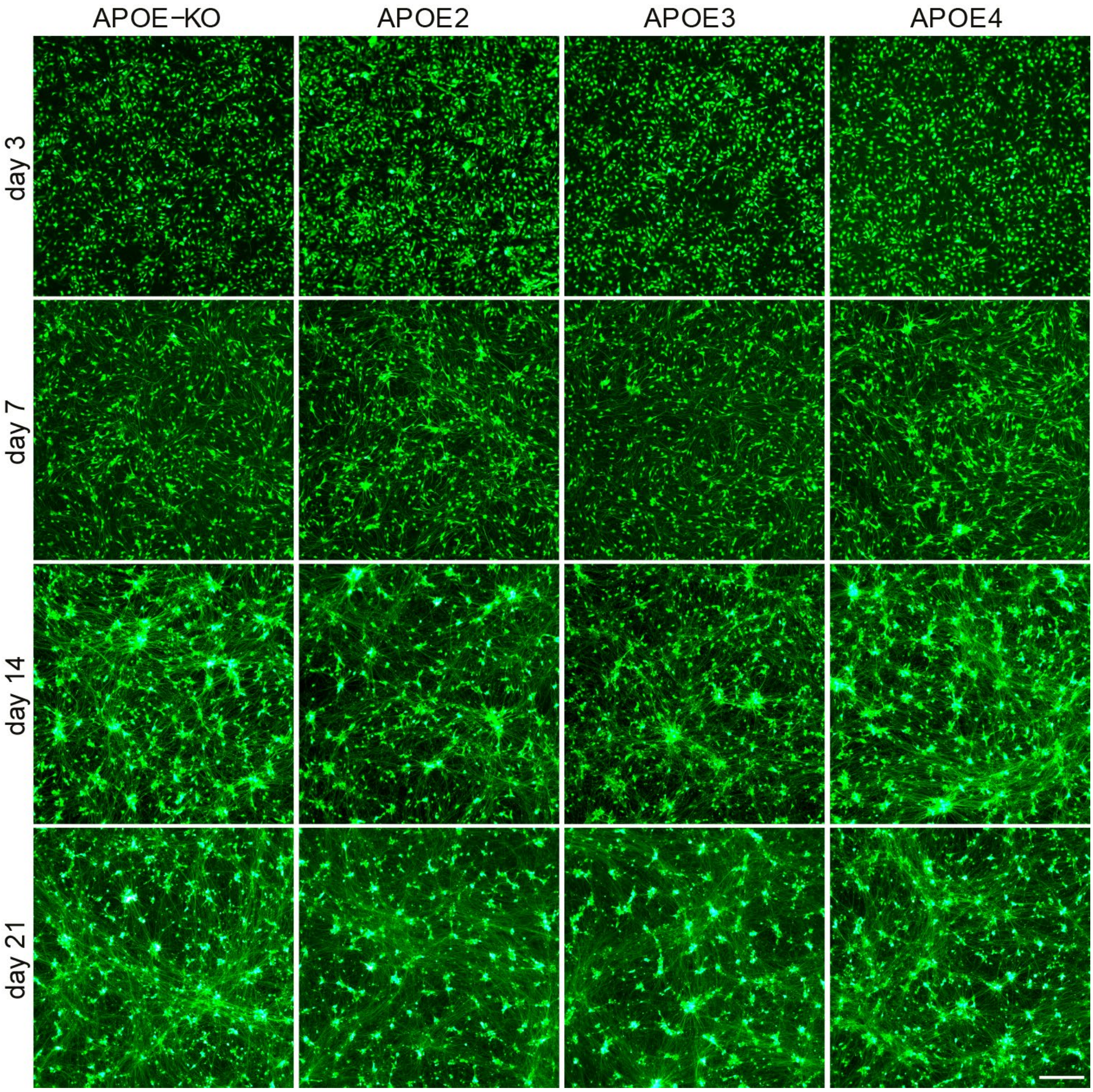

Figure S1: iN cell differentiation over time. Representative fluorescence images of GFP-expressing *APOE*-isogenic iN cells at different time points after induction of differentiation. Scale bar: 50 µm

Table S1: List of qPCR primer.

**GAPDH**	FWD	ACC ACA GTC CAT GCC ATC AC
	REV	TCC ACC CTG TTG CTG TA
**MAP2**	FWD	CTC AGC ACC GCT AAC AGA GG
	REV	CAT TGG CGC TTC GGA CAA G
**OCT4**	FWD	GAG AAG CTG GAG CAA AAC CC
	REV	ACC TTC CCA AAT AGA ACC CCC
**TUBB3**	FWD	GGC CAA GGG TCA CTA CAC G
	REV	GCA GTC GCA GTT TTC ACA CTC
